# *In vitro* activity of bedaquiline against rapidly growing nontuberculous mycobacteria

**DOI:** 10.1099/jmm.0.000537

**Published:** 2017-07-28

**Authors:** Diana A. Aguilar-Ayala, Margo Cnockaert, Emmanuel André, Koen Andries, Jorge A. Gonzalez-Y-Merchand, Peter Vandamme, Juan Carlos Palomino, Anandi Martin

**Affiliations:** ^1^​ Laboratory of Microbiology, Ghent University, Gent, Belgium; ^2^​ Departmento de Microbiología, Escuela Nacional de Ciencias Biológicas, Instituto Politécnico Nacional, Mexico City, Mexico; ^3^​ Pôle of Medical Microbiology, Institute of Experimental and Clinical Research, Université Catholique de Louvain, Brussels Belgium; ^4^​ Janssen Research and Development, Beerse, Belgium

**Keywords:** resistance to bedaquiline, nontuberculous mycobacteria, *atpE* mutation, *Mycobacterium flavescens*, minimum inhibitory concentration, minimum bactericidal concentration

## Abstract

Bedaquiline (BDQ) has been proven to be effective in the treatment of multidrug-resistant tuberculosis. We hypothesized that BDQ could be a potential agent to treat nontuberculous mycobacterial (NTM) infection. The objective of this study was to evaluate the *in vitro* activity of BDQ against rapidly growing mycobacteria by assessing the minimal inhibitory concentration (MIC) and minimal bactericidal concentration (MBC) against 18 NTM strains. For MIC determination we performed the resazurin microtitre assay broth dilution, and for the MBC the c.f.u. was determined. BDQ exhibited a strong inhibitory effect against most NTM tested; however, for some NTM strains the MBC was significantly higher than the MIC. A new finding is that *Mycobacterium flavescens* has a mutation in the gene *atpE* associated with natural resistance to BDQ. These preliminary promising results demonstrate that BDQ could be potentially useful for the treatment of NTM.

The genus *Mycobacterium* comprises more than 150 different species of mycobacteria with the capacity to cause pathogenicity in humans [1]. Most important among these species, due to their airborne transmission and public health implications, are *Mycobacterium tuberculosis* and *Mycobacterium leprae* causing tuberculosis (TB) and leprosy, respectively. Among nontuberculous mycobacteria (NTM), *Mycobacterium avium* and *Mycobacterium abscessus* represent prevalent sources of infection not only in immunocompromised individuals but also in other susceptible populations, such as in cystic fibrosis patients [2]. More recently, other emerging NTM, such as *Mycobacterium chimaera*, have been reported as causes of outbreaks due to heating–cooling devices in surgical rooms [3].

Drug resistance is one of the key issues associated with the current burden of TB around the world, negatively impacting control of the disease [4, 5]. Consequently, efforts have been devoted to the discovery and development of new anti-TB drugs [6]. As a result two new drugs, bedaquiline (BDQ) and delamanid, were recently approved for the treatment of multidrug-resistant TB (MDR-TB) [7, 8]. BDQ has a broad antimycobacterial spectrum and a novel mode of action, inhibiting the ATP synthase [9]. We hypothesized that BDQ could also treat NTM infection.

For infections caused by NTM, combination antimicrobial chemotherapy is the treatment of choice in most cases [10, 11]. Nevertheless, NTM is difficult to eradicate because most of them are naturally resistant to many common antibiotics and in many cases become refractory to the commonly recommended antibiotics [12]. In this context, BDQ has recently been used as off-label for salvage treatment in patients with *Mycobacterium intracellulare* lung disease, with encouraging results [13]. In order to shed light on the conditions and parameters guiding the potential use of BDQ for NTM infections, we evaluated its *in vitro* activity against a panel of rapidly growing NTM reference strains and clinical isolates, and explored the possible correlation of single nucleotide polymorphisms in the target gene and natural resistance to the drug.

Eighteen rapidly growing mycobacterial strains were used in this study (Table 1). Seventeen were obtained from the CCUG collection (http://www.ccug.se) and one strain from the UCL collection in Brussels, Belgium. Strains were cultured on Löwenstein–Jensen medium and the inoculum was prepared in distilled water, adjusted to McFarland 0.5 and diluted 1 : 10 in Mueller–Hinton (MH) broth medium. *Mycobacterium smegmatis* CCUG 28063 was used for quality control since its minimum inhibitory concentration (MIC) for BDQ of 0.015 µg ml^−1^ is well known [9]. To assess whether BDQ had a bacteriostatic or bactericidal effect, we determined the MIC and minimum bactericidal concentration (MBC) using the resazurin microplate assay (REMA) [14, 15]. Briefly, twofold serial dilutions were made in MH in 96-well polystyrene plates. BDQ concentrations were 2.0–0.0035 µg ml^−1^ and each experiment was performed in triplicate. An inoculum equal to McFarland 0.5 diluted 1 : 10 was prepared. Growth controls without drug (positive control), a drug control and a sterile control (negative control) were also prepared for each assay. To prevent evaporation during incubation, 200 µl sterile distilled water was added to all perimeter wells. Plates were sealed and incubated at 37 °C for 3 days before adding 30 µl of 0.01 % resazurin to all wells and incubating for a further 24 h. The MIC was determined as the lowest drug concentration that prevented growth and, therefore, a colour change from blue (oxidized state) to pink (reduced state). MIC values were scored for each isolate tested. The same plates were used for MBC determination. At day 4 of incubation and after the MIC reading, four blue wells were chosen to test the viability of the mycobacteria. One hundred microlitres from each well at the MIC, one concentration higher, and the previous two BDQ dilutions, were transferred to a tube and diluted in sterile distilled water to 10^−3^, 10^−4^ and 10^−5^ and plated in duplicate on Luria broth (LB) agar plates to determine the c.f.u. Also, c.f.u. were determined in duplicate for the positive control diluted 10^−4^, 10^−5^ and 10^−6^. The plates were incubated for 4 days. The percentage of killed bacteria was calculated against the control, and the MBC was defined as the lowest drug concentration that killed 99.9 % of bacteria.

**Table 1.** MIC and MBC range for 18 NTM strains

**EMBL accession number** **for *atpE* sequences**	**Collection number, strain reference**	**Species**	**Bedaquiline**	
			**MIC (µg ml^−1^)**	**MBC (µg ml^−1^)**
LT841272	CCUG 28063, R-50263	*Mycobacterium smegmatis*	0.015	>2
LT841273	CCUG 28060, R-50260	*Mycobacterium phlei*	0.07	>2
LT841274	CCUG 41352, R-52136	*Mycobacterium duvalii*	2	>2
LT841275	CCUG 55442, R-52147	*Mycobacterium cosmeticum*	0.007	0.015
LT841276	CCUG 25238, R-50254	*Mycobacterium mucogenicum*	0.062	>0.5
LT841277	CCUG 37665^T^, R-50255	*Mycobacterium neoaurum*	2	2
LT841278	CCUG 28064, R-50259	*Mycobacterium peregrinum*	0.015	>2
LT841279	CCUG 20999^T^, R-50257	*Mycobacterium parafortuitum*	0.015	>2
LT841280	CCUG 29050, R-50269	*Mycobacterium flavescens*	>2	>2
LT841281	UCL 22248247, R-50337	*Mycobacterum flavescens*	>2	>2
LT841282	CCUG 31556, R-50246	*Mycobacterium fortuitum*	0.031	0.062
LT841283	CCUG 39181, R-52149	*Mycobacterium mageritense*	0.062	>2
LT841284	CCUG 55633, R-52144	*Mycobacterium mageritense*	0.062	>2
LT841285	CCGU 52054, R-52130	*Mycobacterium wolinskyi*	0.031	>2
LT841286	CCUG 41449, R-50243	*Mycobacterium abscessus*	0.062	>2
LT841287	CCUG 27851, R-50244	*Mycobacterium abscessus*	0.25	>2
LT841288	ATCC 14472, R-50274	*Mycobacterium chelonae*	0.062	>2
LT841289	DSM 45524^T^, R-50963	*Mycobacterium franklinii*	0.062	1

For investigation of the mutation in the gene *atpE*, DNA extraction was carried out according to Perez-Martinez *et al.* [16]. Briefly, a loopful of mycobacteria from a Löwenstein–Jensen culture was resuspended in 100 µl Milli-Q water, boiled for 5 min, placed on ice for 10 min, centrifuged at room temperature (13 600 ***g***, 5 min) and the supernatants were used for PCR. The *atpE* gene was amplified using degenerated primers *atpE* forward (degenerated) 5′-TGTAYTTCAGCCARGCSATGG-3′ and *atpE* reverse (degenerated) 5′-CCGTTSGGDABGAGGAAGTTG-3′ [17]. However, if these primers did not amplify *atpE*, a second set of primers was used: *atpE* forward 5′ -TGTACTTCAGCCAAGCGATGG-3′ and *atpE* reverse 5′-CCGTTGGGAATGAGGAAGTTG-3′ [18]. For the degenerated primers, the PCR was run with an initial pre-denaturation at 95 °C for 5 min, followed by 30 cycles of denaturation at 95 °C for 1 min, annealing at 57 °C for 1 min and elongation at 72 °C for 1 min. The reaction was finished with 7 min final elongation at 72 °C. Amplicons were detected by agarose (1.5 %) gel electrophoresis and ethidium bromide staining.

For the second set of primers, the PCR was run with an initial pre-denaturation at 95 °C for 5 min, followed by 30 cycles of denaturation at 95 °C for 1 min, annealing at 62 °C for 1 min and elongation at 72 °C for 1 min. The reaction was finished with 7 min final elongation at 72 °C.

In both cases, identical primers were used for sequencing PCR (BigDye Terminator Sequencing Kit; Applied Biosystems,). Purified products (BigDye XTerminator kit; Applied Biosystems) were sequenced using the ABI Prism 3130 XL Genetic Analyzer (Applied Biosystems). Sequence assembly was performed with BioNumerics v. 7.0 (Applied Maths). Blast sequence software was used to align the wild-type H37Rv *atpE* gene (GenBank accession number NC_000962.3 and GeneID:886937) with the sequences obtained. Nucleotide alignment was done using ClustalX version 2.0 software. This alignment was cleaned with GeneDoc Version 2.7.000 and translated into protein sequences and aligned with mega version 7.0.21 software.

MIC and MBC results are shown in Table 1. For the majority of strains, BDQ had a significantly higher MBC compared to the respective MIC, suggesting a bacteriostatic effect. This occurred for *Mycobacterium smegmatis, M. phlei, M. peregrinum, M. parafortuitum, M. mageritense, M. wolinskyi, M. abscessus* and *M. chelonae*. On the other hand, *M. duvalii* and *M. neoaurum* had a high MIC (2 µg ml^−1^) without *atpE* mutation; however, as the other genes of the ATP synthase operon were not sequenced beside *atpE*, we cannot exclude a mutation in one of the other seven genes of the ATP synthase operon. Interestingly, the BDQ MBC value was almost equivalent to the MIC for *M. cosmeticum* and *M. fortuitum*. BDQ also showed bactericidal activity against *M. mucogenicum* and *M. franklini*, with an MBC of 0.5 and 1 µg ml^−1^, respectively. The MIC of 0.06 µg ml^−1^ for *M. chelonae* is the same as previously described by Huitric *et al*. [17]. In the same study they found a BDQ MIC of 0.13–0.25 µg ml^−1^ for *M. fortuitum* while in this study we found a lower MIC of 0.03 µg ml^−1^ The MIC of BDQ for *M. mageritense* was similar in both studies, at 0.03 (Huitric *et al*. [17]) and 0.06 µg ml^−1^ (this study). On the contrary, *M. flavescens* was completely resistant to BDQ. Three NTM species (*M. xenopi, M. novocastrense* and *M. shimoidei*) with significantly higher MICs for BDQ have been described and are considered naturally resistant to BDQ [17]. Petrella *et al.* [19] reported that *M. xenopi* had a high BDQ MIC (4 µg ml^−1^), most likely attributed to the polymorphism seen at amino acid 63 of AtpE. To investigate whether a similar polymorphism existed and to look into the possible role of gene mutations on the activity of BDQ against NTMs, the gene *atpE* was sequenced in all strains. Fig. 1 shows the amino acid sequence alignment confirming that the degree of identity at the protein level is very high for all NTM tested compared to *M. tuberculosis* H37Rv. An interesting finding of this study is that for *M. flavescens*, as previously reported for *M. xenopi*, *M. shimoidei* and *M. novocastrense*, the alanine at position 63 is replaced by a methionine. In all other known NTM AtpE sequences, this alanine is conserved. We confirmed the presence of this methionine at position 63 by sequencing *atpE* in one clinical *M. flavescens* isolate from St Luc Hospital, Brussels. The presence of this specific mutation is clearly associated with resistance to BDQ resulting in a high MIC. Our data show that the mechanism by which the NTM are inhibited in their growth (as reflected by their MIC) may be different from the mechanism by which they are killed (as reflected by their MBC). BDQ did not show bactericidal activity for the majority of strains tested. However, we did find BDQ bactericidal activity for *M. cosmeticum, M. mucogenicum, M. fortuitum* and *M. franklinii*. This finding should be confirmed with a larger number of clinical isolates. There is still more research to be done to explain why the NTM strains tested are highly sensitive to BDQ and which other factors besides polymorphisms in AtpE may influence the sensitivity of NTM to BDQ. In addition, more research is needed to understand why in some species the MIC and MBC of BDQ are very close and for other species the MBC is much higher than the MIC, and to elucidate the most promising companion drugs.

**Fig. 1.**
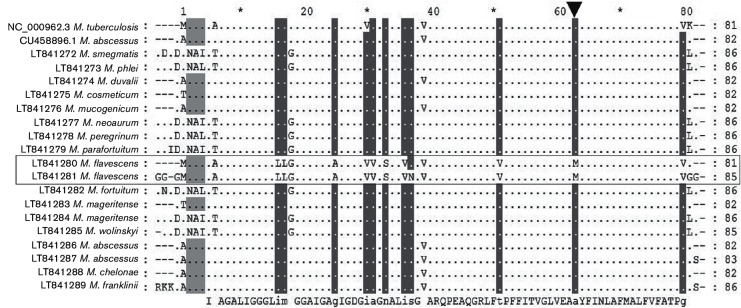
Amino acid alignment of AtpE from selected NTM. Conserved amino acids are represented by dots; only differences among amino acids are shown with letters. Sequences of wild-type *Mycobacterium tuberculosis* H37Rv (GenBank accession number NC_000962.3 and GeneID: 886937) and *Mycobacterium abscessus* ATCC 19977 (GenBank accession number CU458896.1 and GeneID: CAM61534.1) were used as controls. The last line indicates the consensus sequence. Sequences of *M. flavescens* strains R-50269 and R-50337 are highlighted.

In conclusion, to the best of our knowledge, this is the first study to have assessed the MIC and MBC values for BDQ against a large number of rapidly growing NTM. We also described for the first time that *M. flavescens* is naturally resistant to BDQ and its high MIC correlates with the mutation found at amino acid 63 in AtpE (alanine replaced by methionine). However, despite this finding, BDQ exhibited a strong inhibitory effect against all NTM tested, suggesting the potential to treat NTM infections. These preliminary results warrant further research in this area.

## References

[1] Tortoli E (2014). Microbiological features and clinical relevance of new species of the genus *Mycobacterium*. Clin Microbiol Rev.

[2] Martiniano SL, Nick JA, Daley CL (2016). Nontuberculous mycobacterial infections in cystic fibrosis. Clin Chest Med.

[3] Sommerstein R, Schreiber PW, Diekema DJ, Edmond MB, Hasse B (2017). *Mycobacterium chimaera* outbreak associated with heater-cooler devices: piecing the puzzle together. Infect Control Hosp Epidemiol.

[4] Frieden TR, Brudney KF, Harries AD (2014). Global tuberculosis: perspectives, prospects, and priorities. JAMA.

[5] Falzon D, Mirzayev F, Wares F, Baena IG, Zignol M (2015). Multidrug-resistant tuberculosis around the world: what progress has been made?. Eur Respir J.

[6] Ma Z, Lienhardt C, Mcilleron H, Nunn AJ, Wang X (2010). Global tuberculosis drug development pipeline: the need and the reality. Lancet.

[7] Palomino JC, Martin A (2013). TMC207 becomes bedaquiline, a new anti-TB drug. Future Microbiol.

[8] Sotgiu G, Pontali E, Centis R, D'Ambrosio L, Migliori GB (2015). Delamanid (OPC-67683) for treatment of multi-drug-resistant tuberculosis. Expert Rev Anti Infect Ther.

[9] Andries K, Verhasselt P, Guillemont J, Göhlmann HW, Neefs JM (2005). A diarylquinoline drug active on the ATP synthase of *Mycobacterium tuberculosis*. Science.

[10] Esteban J, García-Pedrazuela M, Muñoz-Egea MC, Alcaide F (2012). Current treatment of nontuberculous mycobacteriosis: an update. Expert Opin Pharmacother.

[11] Stout JE, Koh WJ, Yew WW (2016). Update on pulmonary disease due to non-tuberculous mycobacteria. Int J Infect Dis.

[12] Rubio M, March F, Garrigó M, Moreno C, Español M (2015). Inducible and acquired clarithromycin resistance in the *Mycobacterium abscessus c*omplex. PLoS One.

[13] Alexander DC, Vasireddy R, Vasireddy S, Philley JV, Brown-Elliott BA (2017). Emergence of mmpT5 variants during Bedaquiline treatment of *Mycobacterium intracellulare* lung disease. J Clin Microbiol.

[14] Palomino JC, Martin A, Camacho M, Guerra H, Swings J (2002). Resazurin microtiter assay plate: simple and inexpensive method for detection of drug resistance in *Mycobacterium tuberculosis*. Antimicrob Agents Chemother.

[15] Martin A, Camacho M, Portaels F, Palomino JC (2003). Resazurin microtiter assay plate testing of *Mycobacterium tuberculosis* susceptibilities to second-line drugs: rapid, simple, and inexpensive method. Antimicrob Agents Chemother.

[16] Pérez-Martínez I, Ponce-de-León A, Bobadilla M, Villegas-Sepúlveda N, Pérez-García M (2008). A novel identification scheme for genus *Mycobacterium*, *M. tuberculosis* complex, and seven mycobacteria species of human clinical impact. Eur J Clin Microbiol Infect Dis.

[17] Huitric E, Verhasselt P, Andries K, Hoffner SE (2007). In vitro antimycobacterial spectrum of a diarylquinoline ATP synthase inhibitor. Antimicrob Agents Chemother.

[18] Huitric E, Verhasselt P, Koul A, Andries K, Hoffner S (2010). Rates and mechanisms of resistance development in *Mycobacterium tuberculosis* to a novel diarylquinoline ATP synthase inhibitor. Antimicrob Agents Chemother.

[19] Petrella S, Cambau E, Chauffour A, Andries K, Jarlier V (2006). Genetic basis for natural and acquired resistance to the diarylquinoline R207910 in mycobacteria. Antimicrob Agents Chemother.

